# Case Report: Optimized guidewire pacing in transcatheter aortic valve replacement combined with complex PCI in a patient with severe aortic stenosis and regurgitation

**DOI:** 10.3389/fcvm.2025.1515954

**Published:** 2025-07-31

**Authors:** Ruisong Ma, Wang Liao, Lili Zhang, Sheng Wang

**Affiliations:** ^1^ Department of Cardiology, Hainan General Hospital, Haikou, China; ^2^ Department of Cardiology, Hainan Affiliated Hospital of Hainan Medical University, Haikou, China; ^3^ Department of Cardiology, Hainan Clinical Research Center for Cardiology, Haikou, China; ^4^ Department of Cardiology, Hainan Province Clinical Research and Cardiovascular Institute, Haikou, China; ^5^ Department of Cardiology, Hainan Province Clinical Medical Center, Haikou, China

**Keywords:** aortic valve stenosis, coronary artery disease, optimized left ventricular guidewire pacing, transcatheter aortic valve replacement, percutaneous coronary intervention

## Abstract

Left ventricular (LV) guidewire pacing has been proven to be a safe and effective pacing mode for transcatheter aortic valve replacement (TAVR). However, the high pacing voltage threshold and impedance of LV guidewire pacing are potential risks for loss of capture and valve embolization. Moreover, decisions surrounding whether and when to perform percutaneous coronary intervention (PCI) are always heterogeneous in patients with severe aortic stenosis and coronary artery disease. As described in this case report, we attempted an optimized LV guidewire pacing mode with the lowest pacing voltage threshold and impedance, avoiding complications associated with additional vascular access and further reducing TAVR costs. In addition, we successfully performed simultaneous PCI in this patient with a vertically downward orifice of the right coronary artery (RCA), severe calcified stenosis in the RCA, horizocardia, and a dilated ascending aorta. This case report provides new evidence for LV guidewire pacing and the opportunity for PCI in TAVR procedures.

## Introduction

Left ventricular (LV) guidewire pacing has been proven to be a safe and effective alternative strategy to traditional right ventricular (RV) pacing in transcatheter aortic valve replacement (TAVR) (1, 2). Its advantages include (i) reducing the cost of TAVR, (ii) decreasing the procedure duration and fluoroscopy exposure time, and (iii) avoiding the risk of RV perforation and cardiac tamponade. However, the high impedance and pacing voltage threshold of LV guidewire pacing increase the risk of loss of capture and ventricular arrhythmia and can cause fatal complications. To date, only two purpose-designed guidewires for concomitant valve delivery and pacing, the SavvyWire (OpSens Inc., Quebec, Canada) and the Wattson™ temporary pacing guidewire (Teleflex, Inc., Maple Grove, MN, USA), have been approved by the FDA. In most countries and regions of the world, including China, these two guidewires are not available. There is great value in optimizing the traditional guidewire pacing method for TAVR. We aim to propose a superior method in this case report by comparing the impedance and pacing voltage thresholds in different guidewire pacing modes. The patient in this case report also developed severe calcified stenosis in the proximal and middle segments of the right coronary artery (RCA) and a vertically downward orifice of the RCA, with a horizontal aorta (aortic angulation: 67°) and a dilated ascending aorta (diameter 57.3 mm). Concomitant percutaneous coronary intervention (PCI) was performed during the TAVR procedure to address these complications.

## Case presentation

**Figure 1:**
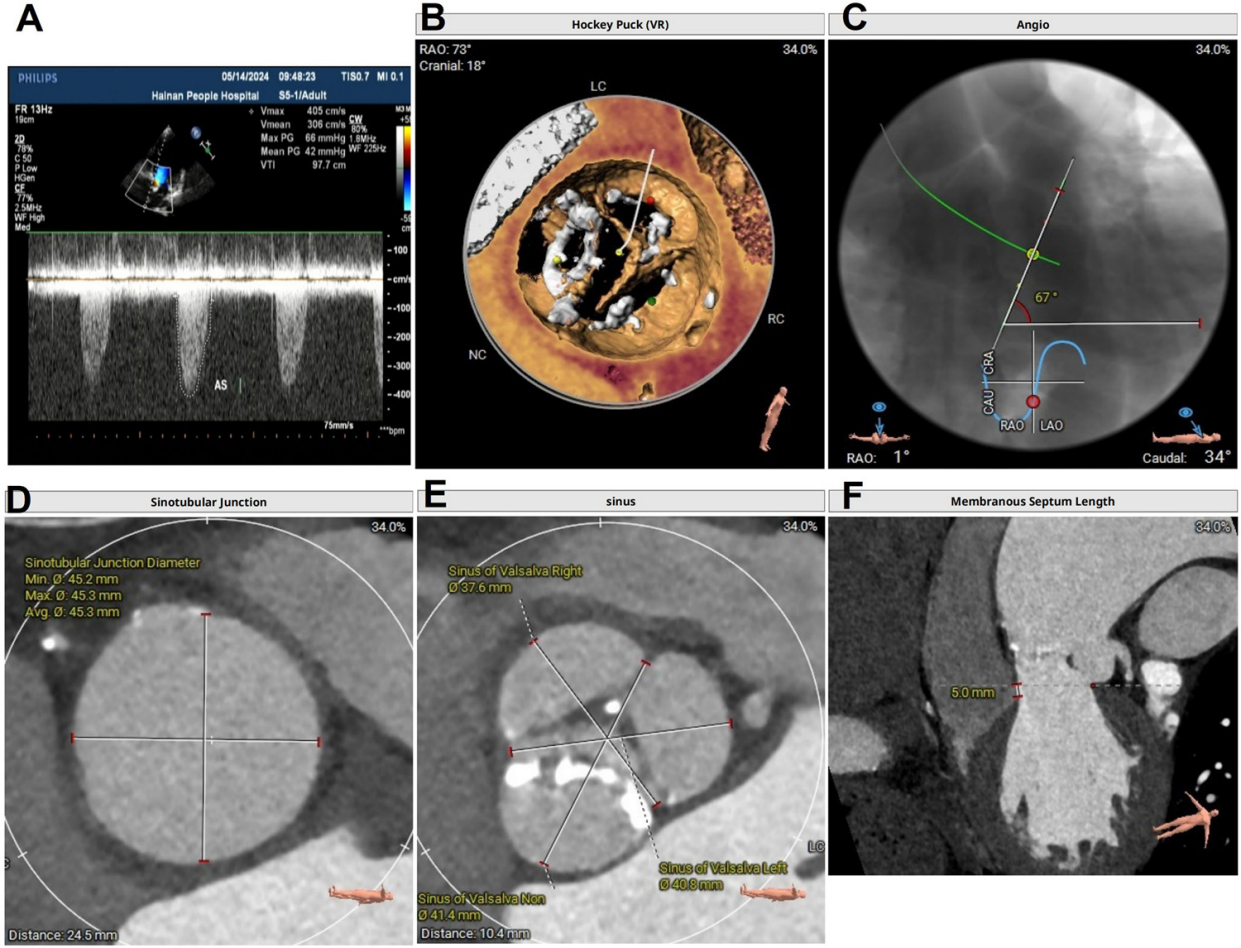
**(A)** Preoperative TTE in systole showing a mean gradient of 42 mmHg and a peak velocity of flow of 4.05 m/s, indicating severe AS; **(B)** preoperative aortic CTA showing a type I functional bicuspid valve; **(C)** preoperative aortic CTA indicating a horizontal heart position, with an angle of 67°; **(D)** a dilated ascending aorta; **(E)** a dilated sinus of Valsalva; and **(F)** the length of membranous septum was 5 mm. TTE, Transthoracic echocardiography; CTA, computed tomography angiography; AS, aortic stenosis.

An 83-year-old male presented with chest tightness, shortness of breath, and cough that had a duration of 2 years. These symptoms had been worsening over the past 2 weeks. He had a history of coronary artery disease (CAD), hypertension, ischemic stroke, frequent atrial premature beats, and surgery for benign prostatic hyperplasia. In addition, his blood pressure was 143/56 mmHg, and his heart rate was 67 bpm. A physical examination revealed a grade 3/6 ejection systolic murmur at the aortic valve auscultation area. Laboratory tests revealed normal creatinine levels (91 μmol/L, normal range 59–104 μmol/L), mildly decreased hemoglobin levels (118 g/L, normal range: 130–175 g/L), and elevated N-terminal probrain natriuretic peptide levels (3,090 ng/L, normal range: <450 ng/L). His initial electrocardiogram (ECG) revealed sinus rhythm without bundle branch block. Preprocedural transthoracic echocardiography (TTE) revealed a normal LV ejection fraction of 51%, a normal LV end-diastolic dimension of 54 mm, severe aortic stenosis (AS) with a peak velocity of 4.05 m/s and a mean gradient of 42 mmHg, and severe aortic regurgitation (AR) (Figure 1A).

Computed tomography (CT) angiography revealed a type 1 bicuspid aortic valve (a valve orifice area of 0.98 cm^2^), with fusion and calcification of the right coronary cusp and left coronary cusp (Figure 1B); a horizontal aorta, with an aortic angulation of 67° (Figure 1C); a dilated ascending aorta, with an average diameter of 57.3 mm (Figure 1D); a dilated sinotubular junction, with an average diameter of 45.3 mm; and a dilated sinus of Valsalva, with an average diameter of 39.9 mm (Figure 1E). The length of the membranous septum was 5 mm (Figure 1F). These anatomical features increased the complexity of the TAVR procedure.

Coronary angiography (CAG) revealed a “shepherd's crook” RCA with a vertically downward orifice and an 80%–90% calcified stenosis in the proximal and middle segments of the RCA (Supplementary Video S1), a dilated ascending aorta with a diameter of 57.3 mm, and horizocardia with an aortic angulation of 67°. It was challenging to perform PCI on this patient.

The patient and his family members declined surgical aortic valve replacement (SAVR), with a EuroSCORE II of 4.2%. Given the severe annular calcification, horizontal cardiac orientation, dilated ascending aorta, and anomalous RCA origin, a balloon-expandable valve would be more suitable. This approach provides superior coaxial alignment and does not compromise the success rate of staged PCI. Owing to cost considerations, they preferred a self-expandable valve over a balloon-expandable valve. Fortunately, the height from the RCA ostium to the annulus measured 22.2 mm, with an RCA leaflet length of 14.5 mm and a self-expandable valve sealing skirt height of 16.5 mm; thus, the patient had a low risk of coronary artery obstruction. Self-expandable valve deployment could have reduced the success rate of PCI of the RCA in this patient's specific aortic sinus anatomy. After a thorough discussion with the multidisciplinary team and discussion with the patient and his family members, the following interventional treatment plan was formulated: first, PCI of the RCA would be performed; if successful, simultaneous TAVR would be performed to mitigate the risk of sudden death associated with staged TAVR; if PCI was unsuccessful, staged TAVR would be performed as an elective procedure. Our extracorporeal membrane oxygenation (ECMO) team and cardiac surgery team would remain on standby.

The area of greatest restriction was located 10 mm above the level of the annulus, where the average diameter was 23.4 mm. Considering the special characteristics of different transcatheter aortic valves (TAVs), a 29# self-expanding VenusA-valve (Venus MedTec Inc., Hangzhou, China), which possesses a more suitable height for the membrane-attached area, was chosen.

## PCI procedure

A 7 Fr Amplatz left II-type guiding catheter was inserted through the right femoral artery but failed to enter the RCA because of the dilated ascending aorta, horizocardia, vertically downward orifice of the RCA, and high blood flow velocity at the aortic orifice. A 0.014 in. hydrophilic Fielder guidewire (Asahi Intecc, Moriyama-Ku, Japan) was inserted into the RCA, and then, a 7 Fr Guidezilla catheter (Boston Scientific, MA, USA) was inserted into the RCA orifice along the Fielder guidewire with inappropriate coaxial engagement (alignment). With the assistance of a 2.0 mm × 20 mm balloon (Boston Scientific, USA), the Guidezilla catheter was advanced into the RCA. After failed attempts, the calcified stenosis was expanded, and a 3.0 mm × 28 mm stent (Boston Scientific, USA) was successfully delivered and implanted in the stenotic segment of the RCA (Supplementary Videos S1–S4).

## TAVR procedure

**Figure 2:**
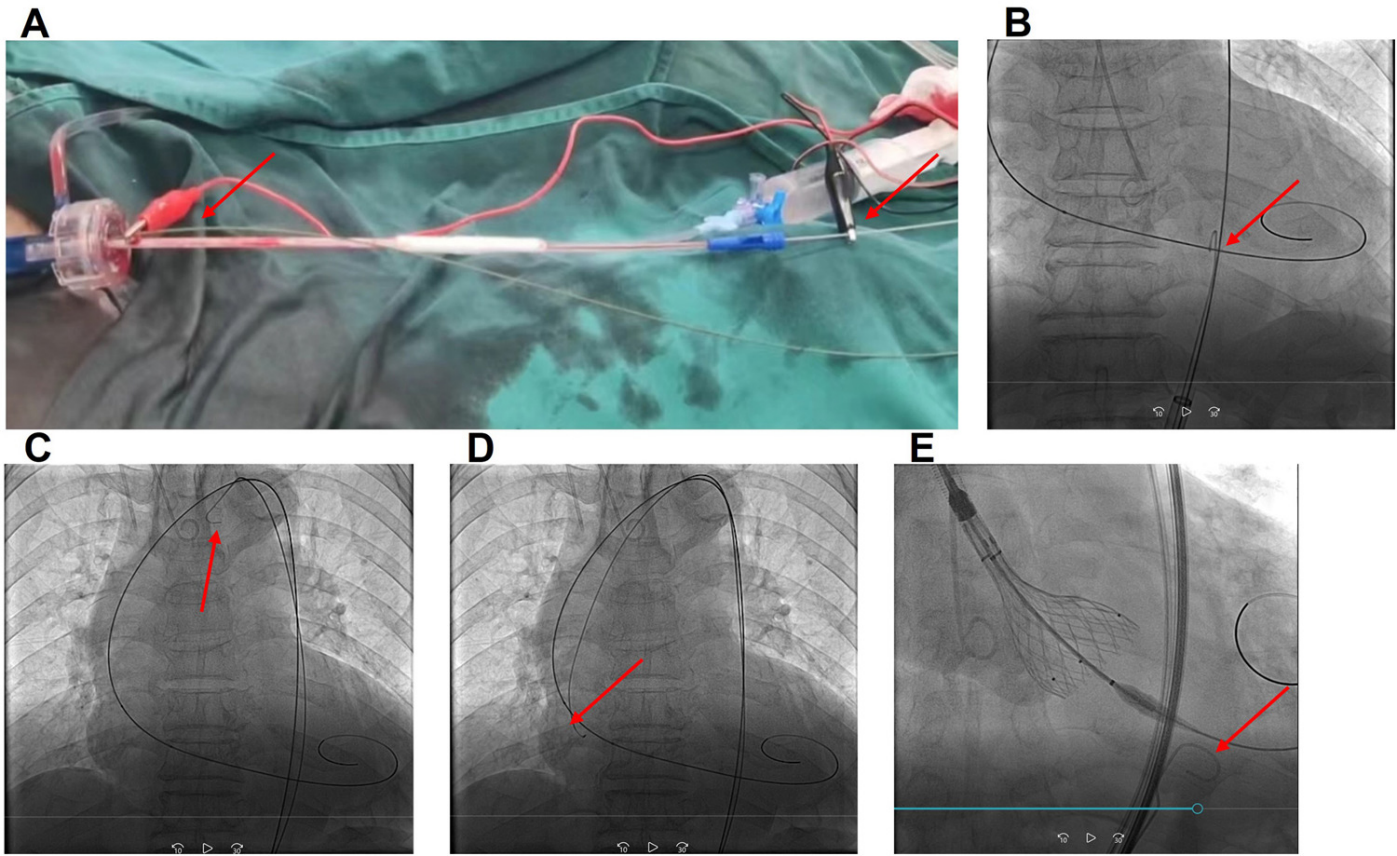
**(A)** The red alligator clip (anode) connected to the end of the J wire, placed in the aorta through a 20 Fr right femoral sheath, and the black alligator clip (cathode) connected to the end of the LV Lunderquist guidewire (cathode) after the balloon or prosthesis was inserted into the annulus; **(B)** J wire advanced in the descending aorta at the same level as the LV Lunderquist guidewire; **(C)** J wire in the arcus aortae; **(D)** J wire in the sinus of Valsalva; and **(E)** OGPM during valve deployment. The red arrows indicate the J wire position. OGPM, optimized LV guidewire pacing mode.

Under general anesthesia, a 6 Fr pigtail catheter was placed at the bottom of the sinus of Valsalva to serve as a landmark through the right radial artery access. A Lunderquist (Cook Medical, Inc., Bloomington, IN, USA) guidewire was preshaped and exchanged into the LV through the right 20 Fr femoral sheath. To ensure good contact with the LV, the Lunderquist guidewire should show dynamic compression during systole under fluoroscopy. A 22 mm × 40 mm balloon (Valgen Medtech, HangZhou, China) was sent into the sinus of Valsalva above the native valve along the Lunderquist guidewire. The black alligator clip was attached to the body of the Lunderquist guidewire (cathode) (Figure 2A).

We tried different anode methods (red alligator): (1) an intravascular J wire (Cordis, Fremont, CA, USA), which was placed within the right 20 Fr femoral sheath; (2) an intravascular J wire, which was placed in the descending aorta, at the same level as the LV Lunderquist guidewire, through the right femoral sheath (Figure 2B); (3) an intravascular J wire placed in the arcus aortae (Figure 2C); (4) an intravascular J wire placed in the sinus of Valsalva (Figure 2D); (5) a subcutaneous needle fixed at the hypogastrium; (6) skin tissue of the lower abdomen; and (7) a grounding pad pasted at the left lateral chest wall.

The impedance and voltage thresholds of different methods were tested by a temporary pacemaker (Medtronic 5318, pulse width: 1.5 ms, Medtronic, Inc., Minneapolis, MN, USA). In method 2, the red alligator clip (anode) was connected to the end of the J wire, which was placed in the descending aorta, at the same level as the LV Lunderquist guidewire, through a 20 Fr right femoral sheath, and the black alligator clip (cathode) was connected to the end of the LV Lunderquist guidewire (cathode) after the balloon or prosthesis was sent into the annulus. This resulted in the smallest impedance (260 Ω) and lowest pacing voltage threshold (3 mV) (Figures 2A,B). The detailed data are presented in Table 1.

**Table 1:** Impedance and pacing voltage threshold of different methods.

Value position	Impedance (*Ω*)	Pacing voltage threshold (mV)
J wire within the sheath	2,120	10
J wire in the descending aorta at the same level as the LV Lunderquist guidewire	260	3
J wire at the arcus aortae	560	6
J wire at the sinus of Valsalva	420	6
Subcutaneous needle	1,220	9
The teeth of alligator clip directly clamped onto the skin	1,260	9
Grounding pad	850	7

On the basis of these tests, method 2 was chosen and defined by our team as the optimized LV guidewire pacing mode (OGPM).

An LV capture check (rate: 120 bpm, voltage: 10 mV, pulse width: 1.5 ms) was performed before balloon dilation. The TAV was advanced and positioned with the assistance of a snare because of the horizontal and dilated aorta. A second check was performed (rate: 120 bpm, voltage: 10 mV, pulse width: 1.5 ms) to confirm complete LV capture. OGPM pacing started at 30 bpm above the baseline heart rate and increased to 180 bpm. The TAV was successfully deployed with one attempt. No conduction problems were found after deployment.

## Discussion

Given the coexistence of severe AS and complicated CAD, with a EuroSCORE II of 4.2%, the rationale for the strategy selected in this case is described below. First, a TAVR + PCI strategy or a SAVR + coronary artery bypass grafting (CABG) strategy was needed. A study revealed that, compared with the TAVR + PCI strategy, the SAVR + CABG strategy resulted in the same incidence of major adverse cardiac events (MACEs) in patients with low-intermediate surgical risk and low-intermediate complex CAD but a lower incidence of MACEs in patients with low-intermediate surgical risk and highly complex CAD at the 30-day follow-up (3). However, TAVR + PCI was associated with a lower incidence of MACEs and mortality in terms of long-term prognosis, although it was associated with a higher incidence of 30-day complications (4). This information, along with the preferences of the patient and his family, made TAVR + PCI more suitable for the patient in our case study. However, more objective studies are needed to help guide treatment selection between TAVR + PCI and SAVR + CABG.

Second, whether and when to perform PCI is a complex decision. The treatment effects of PCI on short-term and long-term prognoses are likely to be heterogeneous (5, 6). Some scholars do not support routine pre-TAVR revascularization with PCI because of an increased risk of life-threatening bleeding and no change in the 30-day and 1-year outcomes (7). The majority of studies recommend that the decision surrounding whether PCI should be performed should be individualized (6, 8). The patient in our report had aggravated chest tightness; severe stenosis in the proximal and middle segments of the RCA; heart failure; anemia, with no history of gastrointestinal bleeding or hemorrhagic stroke; and normal renal function. Complex and high-risk PCI procedures are safe in patients with severe AS and concomitant complex CAD (9). In addition, it was feasible for our heart team to manage this complex and high-risk PCI treatment. Thus, we reached a consensus to perform PCI treatment. Although concomitant PCI has the same early and long-term outcomes as staged PCI (10), it reduces rates of renal dysfunction and costs (8) and has a higher success rate for RCA angiography or PCI (11). This patient developed a vertically downward orifice of the RCA, with horizocardia and a dilated ascending aorta. It is easy to anticipate that staged PCI has a lower success rate than concomitant PCI does. Compared with simultaneous TAVR, staged TAVR may increase the patient's risk of heart failure or mortality due to aortic stenosis; however, the RCA PCI procedure was successful. Ultimately, we opted to perform concomitant PCI and TAVR on the patient. The results of repeated renal function tests 2 days after TAVR were not significantly different from the preoperative values (86 vs. 91 μmol/L, normal range 59–104 μmol/L).

Compared with RV pacing, LV guidewire pacing reduces the TAVR procedure time, cost, and fluoroscopy exposure time, with similar safety and efficacy (12, 13). A high pacing voltage threshold and impedance are potential risks for loss of capture and valve embolization, which might cause fatal complications. Utilization of LV guidewire pacing during balloon inflation increases the rate of loss of LV capture from 0.5% to 4% (14), which may be similar to the results of the valve deployment procedure. In the patient in our report, our heart team attempted an OGPM, which resulted in low impedance (260 Ω) and pacing voltage threshold (3 mV). The underlying mechanisms might be associated with the short distance between the cathode and the anode in the OGPM and the excellent electrical insulation provided by the 20 Fr sheath. OGPM seems to be safer and more efficient than traditional LV guidewire pacing. Second, the J wire was advanced into the descending aorta through the ipsilateral femoral artery in the OGPM, avoiding complications associated with additional vascular access. Third, a J wire catheter was redundant because of the excellent electrical insulation provided by the 20 Fr sheath, further reducing TAVR costs. Thus, OGPM might be a potential optimized LV guidewire pacing mode in TAVR compared with the traditional LV guidewire pacing mode.

In addition, another challenge of the TAVR procedure in the present patient was prosthesis valve crossing and coaxiality due to horizocardia and a dilated ascending aorta. The snare-assisted method was a feasible choice to solve this problem (15). The prosthesis was captured in the distal fifth with a snare at the descending aorta level, maintaining the necessary tension of the snare catheter to optimize prosthesis coaxiality when crossing the aortic arch and aortic valve and ensuring proper valve positioning. After valve deployment, normal images of the aortic valve with no apparent perivalvular leak were observed via TTE and fluoroscopy.

## Conclusions

OGPM might be an optimized method for LV guidewire pacing because of its superior safety and efficacy profile, avoidance of complications associated with additional vascular access, and low TAVR costs.

## Limitations

The findings of this study must be considered in light of the following limitations. (1) For ethical reasons, we did not compare the efficacy between OGPM and RV pacing because of concerns regarding imposing additional costs on the patient. It is generally accepted that traditional LV pacing and RV pacing have comparable safety and efficacy. Compared with traditional lead pacing, OGPM appears to have lower thresholds and impedances. However, further research is needed to validate the safety and efficacy of OGPM vs. RV pacing. (2) The patient develops aortic stenosis combined with regurgitation. TTE carries a potential risk of overestimating stenosis severity when assessed solely by pressure gradient and flow velocity. Measuring the valve orifice area using the continuous-wave Doppler method provides a more objective assessment of stenosis severity. Therefore, we have included the valve orifice area measured by aortic CTA for comprehensive evaluation.

## Data Availability

The original contributions presented in the study are included in the article/Supplementary Material, and further inquiries can be directed to the corresponding author.

## References

[1] Yamashita Y Sicouri S Baudo M Dokollari A Rodriguez R Gnall EM Impact of coronary artery disease and revascularization on outcomes of transcatheter aortic valve replacement for severe aortic stenosis. Cardiovasc Revasc Med. (2024) 68:8–14. 10.1016/j.carrev.2024.05.003 38719630

[2] Kleczynski P Dziewierz A Socha S Rakowski T Daniec M Zawislak B Direct rapid left ventricular wire pacing during balloon aortic valvuloplasty. J Clin Med. (2020) 9:1017. 10.3390/jcm9041017 32260289 PMC7230545

[3] del Portillo JH Farjat-Pasos J Galhardo A Avvedimento M Mas-Peiro S Mengi S Aortic stenosis with coronary artery disease: SAVR or TAVR—when and how? Can J Cardiol. (2024) 40:218–34. 10.1016/j.cjca.2023.09.023 37758014

[4] Morgan AH Zitsch RP. Recurrent respiratory papillomatosis in children: a retrospective study of management and complications. Ear Nose Throat J. (1986) 65:19–28. 3769839

[5] Takasaka K. Daily travel of Sundanese mothers in an agricultural community of West Java: evidence of restriction by childcare. J Hum Ergol (Tokyo). (1986) 15:139–45. 3598183

[6] Stefanini GG Gitto M. The dilemma of CAD in TAVR candidates: useful to find it, useless to treat it? JACC Cardiovasc Interv. (2022) 15:1621–3. 10.1016/j.jcin.2022.07.002 35981835

[7] Altibi AM Ghanem F Hammad F Patel J Song HK Golwala H Clinical outcomes of revascularization with percutaneous coronary intervention prior to transcatheter aortic valve replacement: a comprehensive meta-analysis. Curr Probl Cardiol. (2022) 47:101339. 10.1016/j.cpcardiol.2022.101339 35908687

[8] Tran Z Hadaya J Downey P Sanaiha Y Verma A Shemin RJ Staged versus concomitant transcatheter aortic valve replacement and percutaneous coronary intervention: a national analysis. JTCVS Open. (2022) 10:148–61. 10.1016/j.xjon.2022.02.019 36004248 PMC9390561

[9] Kodra A Basman C Pirelli L Wang D Rahming H Chaudhary R Short-and mid-term outcomes of complex and high-risk versus standard percutaneous coronary interventions in patients undergoing transcatheter aortic valve replacement a total of 276 consecutive patients with severe. Index J Invasive Cardiol. (2023) 35:92–8. 10.25270/jic/22.00254 36525541

[10] Zhang X Geng W Yan S Zhang K Liu Q Li M. Comparison of the outcomes of concurrent versus staged TAVR combined with PCI in patients with severe aortic stenosis and coronary artery disease: a systematic review and meta-analysis. Coron Artery Dis. (2024) 35:481–9. 10.1097/mca.0000000000001373 38682469

[11] Diaz MA Patton M Valdes P Vieira JL Rmeileh A Macedo FY. A systematic review and meta-analysis of delayed coronary artery access for coronary angiography with or without percutaneous coronary intervention (PCI) in patients who underwent transcatheter aortic valve replacement (TAVR). Cardiovasc Intervention Ther. (2022) 37:167–81. 10.1007/s12928-020-00753-4 33453034

[12] Faurie B Souteyrand G Staat P Godin M Caussin C Van Belle E Left ventricular rapid pacing via the valve delivery guidewire in transcatheter aortic valve replacement. JACC Cardiovasc Interv. (2019) 12:2449–59. 10.1016/j.jcin.2019.09.029 31857014

[13] Savvoulidis P Mechery A Lawton E Ludman PF Sharma H Thompson S Comparison of left ventricular with right ventricular rapid pacing on tamponade during TAVI. Int J Cardiol. (2022) 360:46–52. 10.1016/j.ijcard.2022.05.035 35597495

[14] Tanaka M Yanagisawa R Yashima F Arai T Watanabe Y Naganuma T A novel technique to avoid perforation of the right ventricle by the temporary pacing lead during transcatheter aortic valve implantation. Cardiovasc Intervention Ther. (2021) 36:347–54. 10.1007/s12928-020-00676-0 32474841

[15] Yu J Du R Ding F Zhang R Zhu Z. Case report of snare-assisted coaxiality optimized technique during valve deployment in patient with pure aortic regurgitation. Front Cardiovasc Med. (2024) 11:1383264. 10.3389/fcvm.2024.1383264 38784174 PMC11112030

